# Virtual Reality Experiments on Emotional Face Recognition Find No Evidence of Mood-Congruent Effects

**DOI:** 10.3389/fpsyg.2020.00479

**Published:** 2020-04-09

**Authors:** Lan Zhong, Yamin Wang, Hong Kan, Jinhong Ding

**Affiliations:** Beijing Key Laboratory of Learning and Cognition, College of Psychology, Capital Normal University, Beijing, China

**Keywords:** mood congruity, mood-congruent effect, mood induction, emotional memory, gaze tracking, virtual reality, face recognition, face learning

## Abstract

Mood-congruent effects have been demonstrated many times, but few studies have managed to replicate the effect with natural moods. Additionally, the ecological validity of mood induction and real-time observation deficiency remain unresolved. Using a newly developed, virtual-reality-based eye-tracking technique, the present study conducted real-time observations of mood effects on emotional face recognition with simulated “real-life” pleasant and grisly scenes. In experiment 1, participants performed an emotional face recognition task in both positive and negative virtual reality scenes. The recognition tests and gaze tracking results failed to support mood-congruent effects but did show a mood effect independent of a strong emotional face effect. In experiment 2, participants performed a neutral face recognition task in pleasant and grisly scenes that were matched for arousal levels, and the mood effect disappeared. The results also revealed a robust negativity bias in emotional face recognition, which was found to accompany a mood repair effect.

## Introduction

A mood-congruent effect refers to the psychological phenomenon in which the processing of an emotional stimulus is enhanced when its valence matches one’s current mood state (Bower, 1981). Research examining mood congruity is fundamental for the elucidation of how mood affects emotional memory, which may help to reveal the mechanisms of mood processing and the psychopathology of mood disorders.

Although observations of the phenomenon have been replicated many times since Bower’s initial work (Bower, 1981), there is a lack of consensus as to whether mood congruity can be detected with natural moods (Blaney, 1986; Perrig and Perrig, 1988; Forgas et al., 2009). To date, only one study claimed that mood congruity could be observed with natural moods (Mayer et al., 1995), the others failed to replicate the finding (e.g. Hasher et al., 1985; Parrott and Sabini, 1990; Farach et al., 2014), and some studies even demonstrated mood-incongruent effect with natural moods (e.g. Parrott and Sabini, 1990). Whether mood congruity effect could be observed with natural moods remain unresolved. As for existing studies, the difficulties that researchers have faced in demonstrating mood congruity with natural moods (Hasher et al., 1985; Parrott and Sabini, 1990; Forgas et al., 2009) are consistent with the possibility that traditional laboratory mood induction procedures may differ fundamentally from natural mood formation (Mayer et al., 1995; Eich and Macaulay, 2000). Natural mood congruity ecologically occur in a context that intrinsically embodied a memory task. There are two studies that displayed real-life natural moods by either employing depressed participants or bad weather (Hasher et al., 1985; Farach et al., 2014). However, natural moods induced by these methods are really difficult to manipulate. Studies with good ecological validity and reliability are expected to further examine the problem.

Mood-congruent effects represent one of the interactive patterns between moods and emotional stimuli. Investigating how natural moods interact with emotional stimulus processing requires a well-controlled natural mood induction method, which would meet the criteria of capturing real-time, real-life, and controllable situations. The real-time feature should preclude problems related to a differentiated time course between positive and negative moods (Gray et al., 2002; Goeleven et al., 2007; Toyama et al., 2014). The real-life property should improve ecological validity, and the controllability property refers to the ability to replicate the results across different laboratory settings. To date, however, few studies have demonstrated a mood-congruent effect with natural moods (Hasher et al., 1985; Forgas et al., 2009), although natural moods play an important role in understanding the phenomenon (Parrott and Sabini, 1990; Kwiatkowski and Parkinson, 1994; Mayer et al., 1995). The most challenging task in mood effect studies, especially those conducted with emotional pictures, is how to control the valence and arousal levels of induced moods (Westermann et al., 1996). Together, these two features contribute to the ecological validity of the experiment, while controllability ensures test reliability.

The question of how to obtain direct observations of the interactions between a person’s natural mood and emotional stimuli is another crucial problem in mood effect research. Positive mood has been reported to increase the breadth of attention allocation (Wadlinger and Isaacowitz, 2006; Rowe et al., 2007), and this broadening appears to be implicitly susceptible to distraction (Biss et al., 2010). Therefore, direct and continuous measurement of overt affect-driven visual attention could provide an important supplement to the assessment of behavioral responses to emotional stimuli. A host of studies have demonstrated that eye or gaze tracking can provide a means for observing interactions between natural moods and emotional stimuli in real time (Isaacowitz et al., 2008; Newman and Sears, 2015).

To the best of our knowledge, research has yet to employ a well-controlled natural mood paradigm to observe how mood interacts with emotional stimuli. The development of virtual reality (VR) techniques has made it possible to not only simulate both real-time and “real-life” situations in the laboratory but also to manipulate the valence and arousal levels of the induced moods. In our study, we found that the levels of valence and arousal stimulated in a virtual reality environment (VRE) can be adjusted easily according to participants’ reports. Prior study showed that the arousal level of a VRE was associated with the quality of texture and models (fidelity), while the valence level is mainly dependent on lightness, color, and the pertinent eliciting objects (Marín-Morales et al., 2018). The manipulation of the fidelity and the eliciting stimuli make VR-based mood induction more controllable than traditional mood induction methods. Tentatively, several studies have confirmed the validation of VR-based mood induction (e.g. Felnhofer et al., 2015) and have shown that VRE and “real-life” environments produce similar neural activations in the brain (e.g. Mellet et al., 2010). Moreover, it is also possible to combine eye tracking with VR-based natural mood induction to produce a real-time, real-life observation paradigm. Having considered that eye tracking recordings in a head-mounted helmet actually reflect both eye and head movements, we adopted the term “gaze tracking” rather than “eye tracking” in the present study.

Prior studies testing mood congruity have most frequently ensured the valence level of the induced mood, whereas few studies have controlled or manipulated both valence and arousal levels of the induced moods to observe the possible mood effects (Mayer et al., 1995; Schmid and Mast, 2010). Some recent studies have suggested that arousal but not valence might significantly affect perception and memory processing (Mather and Sutherland, 2011; Mather et al., 2016; Sutherland and Mather, 2017). Logically, valence-based theory tends to support mood-congruent effects, as have most previous studies, whereas valence plus arousal based theory predicts mood effects rather than mood-congruent effects. Based on existing findings, we proposed that VR-based natural moods would affect emotional face recognition and provide evidence of a mood-congruent effect and that the arousal level of the induced mood would partly account for the mood effects. In addition, gaze tracking recordings would provide a direct observation of the interaction between moods and emotional stimuli. Using newly developed eye tracking via a head-mounted helmet, the present study aimed to examine how real-time VR-based natural moods interact with emotional face recognition and how the valence and arousal levels of VR-based natural moods can be controlled in mood research.

To test these hypotheses, we conducted two experiments with a classical face recognition memory task. In experiment 1, the VR scenes and emotional faces were extended to include neutral scenes and neutral faces (valence manipulation) while the arousal level of emotional faces and VREs were further balanced. In experiment 2, only neutral faces were presented in both grisly and pleasant VR scenes to investigate possible mood effects when the arousal levels of the two scenes were comparable.

## Experiment 1

### Methods

#### Participants

A group of 40 participants (18 male, 22 female; mean age, 23 ± 2.5 years; age range, 19–26 years) were recruited from local universities. Eligible participants indicated via the Self-Rating Anxiety Scale (SAS) (Zung, 1971) and the Zung (1965) Self-Rating Depression Scale (SDS) that they did not have serious anxiety problems (SAS cutoff score = 50) or depression (SDS cutoff score = 53) and that they had experience of 3D games. All experimental procedures were approved by the Institutional Review Board of Capital Normal University. The methods were carried out in accordance with the relevant guidelines and regulations. All participants signed an informed consent approved by the Institutional Review Board and were compensated monetarily.

The sample size was determined by three pilot experiments. Experiment A was designed to assess the valence and arousal of VRE simulations and to assess what emotion the negative scene actually induced. Thirty-two participants attended the pilot experiment. The results showed that the levels of valence and arousal met the desired goals on a 7-point scale (valence: *M*_pleasant_ = 4.78, SD = 1.08, *M*_grisly_ = 3.41, SD = 1.04; arousal: *M*_pleasant_ = 3.21, SD = 1.38, *M*_grisly_ = 3.55, SD = 1.38). Just one participant gave a low score for the valence of a pleasant scene (*N*_valence < 3_ = 1), while three participants gave a high score for the valence of a grisly scene (*N*_valence > 5_ = 3). As for emotional face recognition, a large face effect was found on discriminability [*A*′: *F* (1, 31) = 22.52, *p* < 0.001, η*_p_*^2^ = 0.42; hit rate ≈ 0.7]. The other two experiments were conducted to further explore the ways that emotional faces should be displayed in the virtual reality scenes. In addition, the program used to record and analyze gaze tracking data were also tested. Emotional faces were prearranged in scenes in experiment B but were intelligently popped up from where a gaze focused for a while in experiment C. The manipulation also differed slightly: the valence level of a pleasant scene in experiment C was higher than the level in experiment B. Eighty-one healthy participants attended in experiment B (12 male, 20 female; mean age, 22.06 ± 2.05 years; age range, 19–26 years; one male and one female were excluded for sickness and scare) and C (11 male, 29 female; mean age, 23.93 ± 2.99 years; age range, 21–38 years; 7 were excluded for sickness, scare, and equipment failure), respectively. The results showed that fearful faces were encoded more strongly than happy faces in both pleasant and grisly scenes [B: *A*′, *F* (1, 31) = 15.85, *p* < 0.001, η*_p_*^2^ = 0.34; C: *A*′, *F* (1, 39) = 38.41, *p* < 0.001, η*_p_*^2^ = 0.50] and that the number of fixations and participants’ total viewing times were greater for happy faces than for fearful faces in both scenes [B: NF, *F* (1, 31) = 4.01, *p* = 0.054, η*_p_*^2^ = 0.12; C: NF, *F* (1, 39) = 5.21, *p* = 0.028, η*_p_*^2^ = 0.12]. The results of the two experiments fail to support the assumption of a mood-congruent effect with natural moods. Valence and arousal assessments showed that improved fidelity and removal of the perimeter wall increased the valence level and reduced the arousal level of the pleasant scene. The sample size in the current study was thus determined by the pilot experiments (hit rate = 0.7, *f*^2^ = 0.25, *n* = 24).

#### Apparatus

The experiment was performed with a VR system consisting of an Oculus Rift DK2 head-mounted VR helmet (Oculus, Irvine, CA, United States; 100° horizontal field of view; resolution 960 × 1080 pixels per eye; refresh rate, 75 Hz; and delay, 2–3 ms) installed with an SMI eye-movement recording subsystem (refresh rate 75 Hz; gaze error: 0.5 of the visual field; and camera-integrated calibration) and an infrared tracking subsystem comprised of eight ART Track 5 intelligent tracking cameras (refresh rate, 300 Hz) covering a 6-m^2^ floor area and a central controller running DTrack2 software (ART Technology Co., Weilheim, Germany).

The virtual reality environment (VRE) was presented on the head-mounted helmet, which was tracked by the infrared tracking subsystem via a pair of glasses with six passive markers. Sounds were presented via a Wharfedale Pacific Evolution 40 stereo subsystem (Wharfedale, Britain). Notably, the real-time refresh rate recorded during the experiment was ∼79 Hz.

The data were processed at an HP workstation (CPU E5-2667 3.20 GHz, RAM 56.0 GB, GPU NVIDIA Quadro K6000). The software used included 3dsmax 2012 (Autodesk, San Rafael, CA, United States) for modeling, Photoshop CS6 (Adobe Systems Inc., San Jose, CA, United States) for textures, and Unity3d (Unity Technologies SF, Copenhagen, Denmark) for VR scenery and interaction.

#### Materials

Materials used consisted of questionnaires, VREs, and stimuli. Depression was assessed using the 20-item SDS (Zung, 1965), and anxiety problems was assessed using the 20-item the SAS (Zung, 1971), while participants rated their moods via a series of unipolar 7-point scales for valence and arousal level (1 = “not at all;” 4 = “moderately;” 7 = “extremely”).

##### VREs

**FIGURE 1:**
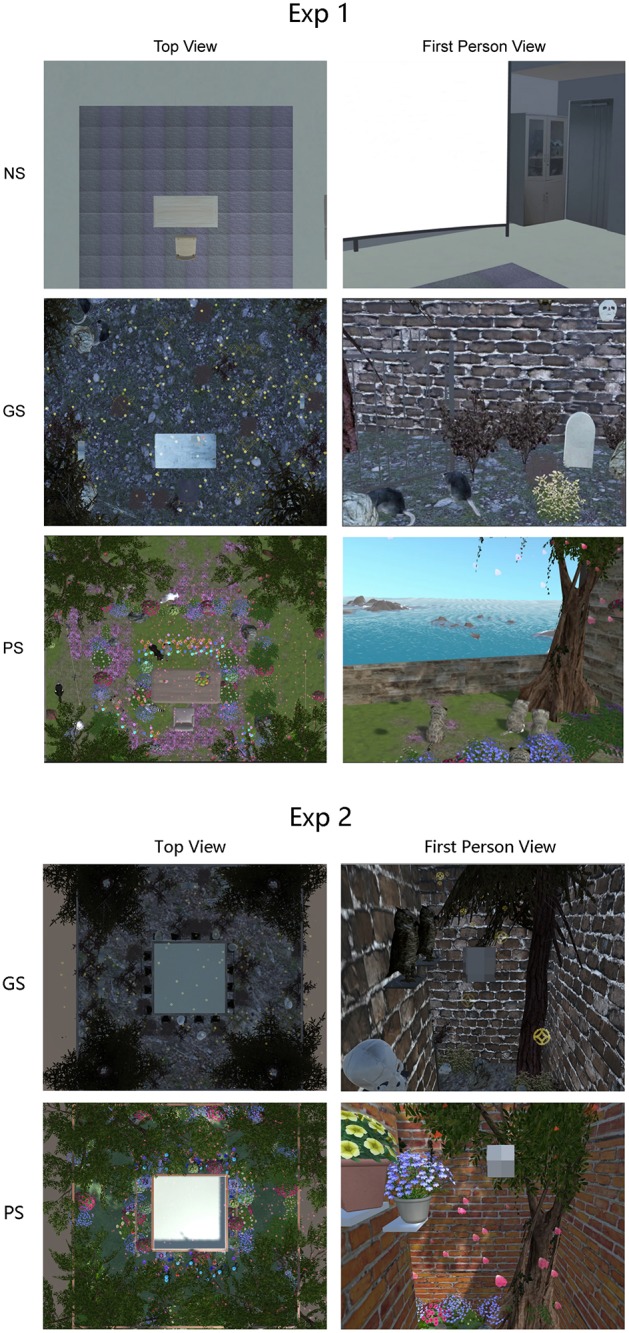
Virtual reality environments (VREs) used in experiments 1 and 2. NS, neutral scene; GS, grisly scene; PS, pleasant scene.

For this experiment, we created mood induction VREs (neutral, pleasant, or grisly scene) in our virtual reality lab using Unity game development software (Unity Technologies, Inc., San Francisco, CA, United States), and the sizes of all three VREs were matched (4.8 m^2^ tracking area monitored with an ART tracking system). The neutral scene was a simulation of our virtual reality lab (Figure 1). In addition to mood induction VREs, a VR testing room (1.5 × 1.5 × 2 m) was located at the center of the tracking area with a gray ceiling and interior walls during testing time. The VR testing room was separated from the learning scene.

For the pleasant VRE, a garden was created in the square learning area (the same size of our lab) with red brick perimeter walls and a blue skybox over the area. One of the walls was deleted so that the square area faced the sea. The garden had a rich variety of beautiful flowers, while timber palisades were arranged along a grass garden path. There was a beautiful big tree at each corner of the garden, and several stones were placed under the trees. In addition, four running kittens, two beautiful flying birds, and the sound of the sea were added to the scene to induce happy mood, as shown in Figure 1. Rose petals drifted down from the sky as the sound of ocean waves and birds was played.

For the grisly VRE, the red brick walls were replaced with drab gray walls. The four trees were made invisible with spider webs emanating from their locations and the walls. Withered grass, tombstones, black iron railings, and skulls were arranged along the path in place of flowers, timber palisades, and stones. Several owls stood on the walls, iron railings and tombstones, and some gray-colored rats with one red eye moved about on the floor. The sound of rats played, and hell money (the money used by ghosts in the hell in Chinese folktales) fell from the sky instead of rose petals. Two flying bats were placed in the grisly scene to increase the arousal level of fear.

By these designs, we intended to match the valence and arousal levels of the neutral, pleasant, and grisly VREs: valence_pleasant_ > valence_neutral_ > valence_grisly_; arousal_grisly_ > arousal_pleasant_ > arousal_neutral_.

##### Facial image

The facial images used in the experiment consisted of 48 fearful faces, 48 happy faces, and 48 neutral faces, which were selected from Luo’s face database (Gong et al., 2011) and the Taiwanese Facial Expression Image Database (TFEID) (Chen and Yen, 2007). Welch’s ANOVA showed a main effect of emotional face on valence [*F* (2, 91.47) = 637.42, *p* < 0.001]. Games–Howell post hoc testing showed that valence was rated higher for neutral faces than for fearful faces and rated lower for neutral faces than for happy faces [difference_neutral – fearful_ = 1.45, *p* < 0.001, SE = 0.08, 95% CI = (1.25, 1.64); difference_neutral – happy_ = −2.26, *p* < 0.001, SE = 0.10, 95% CI = (−2.50, −2.02); difference_happy – fearful_ = 3.71, *p* < 0.001, SE = 0.10, 95% CI = (3.46, 3.96)]. Welch’s ANOVA showed a main effect of scene on arousal [*F* (2, 88.35) = 1,212.34, *p* < 0.001]. Games–Howell post hoc testing indicated that arousal was balanced between happy faces and fearful faces [difference_happy – fearful_ = −0.17, *p* = 0.22, SE = 0.10, 95% CI = (−0.42, 0.07); difference_neutral – happy_ = −3.26, *p* < 0.001, SE = 0.09, 95% CI = (−3.47, −3.05); difference_neutral – fearful_ = −3.44, *p* < 0.001, SE = 0.08, 95% CI = (−3.64, −3.24)]. Thirty-six participants assessed the valence and arousal of the selected faces, and the results are shown in Table 1.

**TABLE 1:** Assessments of faces and scenes for experiment 1, (SD).

Parameter	Face		
	
	Fearful	Happy	Neutral
Valence			
Female	3.19 (0.42)	6.78 (0.56)	4.74 (0.33)
Male	3.18 (0.44)	7.01 (0.58)	4.53 (0.39)
**Arousal**			
Female	6.34 (0.39)	6.23 (0.50)	2.86 (0.32)
Male	6.36 (0.58)	6.13 (0.54)	2.97 (0.29)

**Parameter**	**Scene**		
	
	**Grisly**	**Pleasant**	**Neutral**

Valence	2.90 (1.34)	4.97 (0.97)	3.93 (0.86)
Arousal	4.46 (1.10)	3.74 (1.14)	3.11 (1.15)

*Faces were assessed on a 9-point Likert scales pre-experiment; scenes were evaluated on 7-point Likert scales during experiment.*

Although sadness is frequently a target emotion in mood congruity studies, here, we used a grisly VRE to induce a fearful mood. We did so because we found that we could induce fear more reliably than sadness in VRE pilot experiments. That is, healthy volunteers were more likely to say a “sad” VRE was grisly (or scary) rather than sad.

#### Design

**FIGURE 2:**
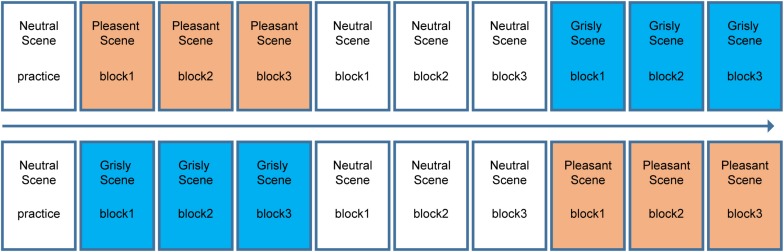
Block design for experiment 1. Top and bottom shows the block order for each half of the participants.

The experiment had a 3 × 3 within-subject design [(mood induction scene in learning phase: pleasant vs. grisly vs. neutral) × (facial emotion: happy vs. fearful vs. neutral)]. A classical recognition memory task used in mood congruity research was used to examine the effects of naturally induced moods on emotional face recognition. Notably, the neutral scene was always placed in the middle, while the order of the pleasant and grisly scenes were balanced between participants. The neutral scene and neutral faces were included to assess whether one or both of the induced moods would influence emotional face recognition. The scene order (pleasant then grisly or vice versa) was pseudo-random across subjects, as summarized in Figure 2. At the end of each block, there was a face recognition test. The 16 test faces in each block consisted of 8 target faces and 8 new faces. Thus, in total, 48 faces were viewed within each set of scene tests (neutral, pleasant, and grisly). The sex and facial emotion of the face images were counterbalanced across all learning and test blocks within each emotional scene by assigning the participants randomly to one of eight emotional face sets, with each face set corresponding to one of eight (2 × 2 × 2) permutations [(scene order) × (scene: pleasant vs. grisly) × (phase: learning vs. testing)].

#### Procedure

The whole experiment consisted of a questionnaire phase, a learning phase, and a testing phase. In the questionnaire phase, participants were asked to answer two questions by telephone to rule out serious motion sickness problems or rodent phobia. When they came to the laboratory, they were asked to complete an SDS questionnaire to rule out depression and an SAS questionnaire to rule out anxiety problems. Mood was assessed during the learning and testing phases. All participants were told that they could ask for a pause and withdraw from the experiment if they felt uncomfortable.

Before the mood induction phase, a practice block was administered to familiarize participants with the buttons. In the learning phase, participants were instructed to view a virtual photo album in which fearful, happy, or neutral face photos were included. They were allowed to view the photo albums freely while sitting in the virtual area facing the blue sea in a beautiful yard (pleasant VRE) or facing the fearful wall in a grisly black graveyard (grisly VRE). They pressed the left bumper button of a wireless joystick to let the face disappear when they felt that they had memorized each face. To ensure sufficient learning time, there was a 500-ms minimal accumulated target-face fixation time before the left bumper button became functional. After learning a full set of eight faces, the participants were instructed to sit in the testing room for the testing phase and end the music.

After entering the testing room, participants were instructed to press the buttons to rate moods. Immediately thereafter, two 7-point scales appeared on the testing room wall one at a time. Participants reported the valence and arousal level that they had just felt in the corridor by selecting a number button on the wall. A random number then appeared, and participants were asked to subtract 7 from it, report the result loudly, and then continue to subtract 7 from the result just reported. The loop calculation lasted for 30 s. A face recognition test followed the calculation, in which the participants were instructed to recognize the face appearing on the wall. They initiated the test by pressing the left bumper button, after which the test began with a 500-ms fixation cross. Subsequently, a face was presented on the wall for a maximum of 1,000 ms. It disappeared when the participant pressed the “X” or “B” button to identify a learned or new face, respectively. All 16 faces (8 learned and 8 new) were randomly displayed, and the responses were recorded. The procedure was identical for all blocks.

#### Data Analysis

##### Gaze tracking records

To record gaze movements during emotional face learning, 460 cell detectors (20 × 23, each measuring 1 × 1 cm) in a virtual reality world were created on each face photo. Each cell detected and recorded the time that the participant’s gaze was on and off the cell. The recorded distance between the camera and face photos ranged from 87 to 205 cm (146 ± 59 cm), resulting in a cell visual angle in the range of 0.28–0.99°. Fixation was defined as the interval in which a gaze was focused within three cells for ≥100 ms (Isaacowitz et al., 2008). Therefore, we combined the cells that satisfied three conditions: consecutive, within three cells, and an interval time <600 ms. The combined and the uncombined cells that participants focused on for more than 100 ms were then defined as a fixation. Based on this definition, two gaze movement indices were computed for each of the nine experimental conditions [(scene: pleasant vs. grisly vs. neutral) × (emotional face: happy vs. fearful vs. neutral)]. The gaze movement indices were the number of fixations (NFs) and mean fixation duration (MFD). Mean total viewing time (MTVT) and mean gaze movement including eye saccade speed (SPEED) were also computed. MTVT was the average time that participants viewed a face under one of the four experimental conditions. SPEED was a rough measurement showing how fast the participants moved their gaze around a facial image while studying it. We computed the cumulative gaze movement distance and all intervals between continuous cell recordings on the original database (no fixations were combined), then divided that total distance by the total interval to obtain a mean gaze movement speed. To avoid artifacts due to anomalous behaviors, such as a few participants occasionally moving their gaze away from the target for internal or personal reasons, intervals >3 s were deleted with the corresponding distance recordings when computing SPEED. Because descriptive measurements can provide holistic information about one’s attention, heat maps were created for each condition with a program written specifically for this experiment, and the fixation areas in each condition were computed for each participant.

##### Statistics

Results were compared across scenes and conditions with analyses of variance (ANOVAs), followed by Games–Howell or least significant difference (LSD) post hoc tests. Mean differences were reported with standard errors (SEs) and 95% confidence intervals (CIs). Face recognition performance results were subjected to simple effects analyses. Correlation analysis among gaze movement indices and discriminability variables were performed with bivariate correlation tests. All statistical analyses were performed using SPSS 22.0.

Hit rates and false alarm (FA) rates for recognition memory were recorded in the experiment. Discriminability was computed with *A*′ and *Pr* because a hit rate of 1 existed (Feenan and Snodgrass, 1990; Donaldson, 1992; Ochsner, 2000). To observe possible judgment bias in recognition memory, $B_{D}^{{}^{\prime \prime }}$ and *Br* were also computed.

### Results

#### Mood Induction

The mood assessment results for the pleasant and grisly scenes are shown in Table 1. Welch’s ANOVA showed a main effect of scene on valence [*F* (2, 75.90) = 32.20, *p* < 0.001]. Games–Howell post hoc testing showed that valence was rated higher for the neutral scene than for the grisly scene and rated lower for the neutral scene than for the pleasant scene [difference_neutral – grisly_ = 1.03, *p* < 0.001, SE = 0.25, 95% CI = (0.43,1.64); difference_neutral – pleasant_ = −1.03, *p* < 0.001, SE = 0.21, 95% CI = (−1.52, −0.54); difference_pleasant – grisly_ = 2.07, *p* < 0.001, SE = 0.26, 95% CI = (1.44, 2.70)]. A one-way ANOVA showed a main effect of scene on arousal [*F* (2,117) = 14.31, *p* < 0.001, η*_p_*^2^ = 0.20]. LSD post hoc testing indicated that arousal was rated lower for the neutral scene than for the grisly scene [difference_neutral – grisly_ = −1.35, *p* < 0.001, SE = 0.25, 95% CI = (−1.85, −0.85); difference_neutral – pleasant_ = −0.63, *p* = 0.014, SE = 0.25, 95% CI = (−1.13, −0.13); difference_pleasant – grisly_ = −0.72, *p* = 0.005, SE = 0.25, 95% CI = (−1.22, −0.22)]. These results show that each VRE scene was able to induce its intended mood at the required levels of valence and arousal.

#### Hit and FA Rates

A 3 × 3 ANOVA [(scene: pleasant vs. grisly vs. neutral) × (emotional face: happy vs. fearful vs. neutral)] showed a marginal effect of emotional face on hit rate [*F* (2, 78) = 2.59, *p* = 0.081, η*_p_*^2^ = 0.062]. A pairwise comparison analysis suggested that hit rates were higher for fearful faces than for neutral faces [difference_fearful – neutral_ = 0.043, *p* = 0.032, SE = 0.019, 95% CI = (0.004, 0.082)]. A 3 × 3 ANOVA [(scene: pleasant vs. grisly vs. neutral) × (emotional face: happy vs. fearful vs. neutral)] revealed a main effect of emotional face on FA rates [*F* (2, 78) = 9.81, *p* < 0.001, η*_p_*^2^ = 0.20] and also a main effect of scene on FA rates [*F* (2, 78) = 3.66, *p* = 0.038, η*_p_*^2^ = 0.086]. A pairwise comparison analysis indicated that FA rates were higher for happy faces than for fearful faces [difference_happy – fearful_ = 0.082, *p* < 0.001, SE = 0.020, 95% CI = (0.043, 0.122)] and that FA rates were higher for happy faces than for neutral faces [difference_happy – neutral_ = 0.052, *p* = 0.003, SE = 0.017, 95% CI = (0.018,0.086)]. As for scene effects, Greenhouse–Geisser revealed that FA rates were higher for the grisly scene than for the neutral scene [difference_grisly – neutral_ = −0.053, *p* = 0.002, SE = 0.016, 95% CI = (0.020, 0.086)].

#### Discriminability

The *A*′ and *Pr* values computed for the participants are presented in Table 2. A 3 × 3 ANOVA [(scene: pleasant vs. grisly vs. neutral) × (emotional face: happy vs. fearful vs. neutral)] showed a main effect of emotional face on *A*′ [*F* (2, 78) = 10.11, *p* < 0.001, η*_p_*^2^ = 0.21] and on *Pr* [*F* (2, 78) = 11.09, *p* < 0.001, η*_p_*^2^ = 0.22], suggesting that fearful faces were encoded more strongly than happy and neutral faces. ANOVA also showed a main scene effect on *A*′ [*F* (2, 78) = 3.93, *p* = 0.034, η*_p_*^2^ = 0.092] and on *Pr* [*F* (2, 78) = 4.70, *p* = 0.012, η*_p_*^2^ = 0.11], suggesting that emotional faces were encoded more strongly in the neutral scene than in the pleasant and grisly scenes.

**TABLE 2:** Emotional face recognition results for experiment 1, (SD).

Condition	Raw measure		Discrimination		Bias	
			
	Hit rate	FA rate	*A*′	*Pr*	$B_{D}^{{}^{\prime \prime }}$	*Br*
GSFF	0.78 (0.21)	0.18 (0.17)	0.84 (0.12)	0.53 (0.23)	0.06 (0.58)	0.47 (0.26)
GSHF	0.76 (0.19)	0.26 (0.23)	0.80 (0.13)	0.45 (0.25)	0.001 (0.56)	0.49 (0.23)
GSNF	0.76 (0.19)	0.19 (0.19)	0.83 (0.13)	0.51 (0.24)	0.12 (0.56)	0.45 (0.24)
PSFF	0.80 (0.15)	0.15 (0.17)	0.86 (0.09)	0.58 (0.19)	0.13 (0.56)	0.44 (0.25)
PSHF	0.77 (0.16)	0.21 (0.21)	0.82 (0.12)	0.50 (0.23)	0.06 (0.56)	0.46 (0.24)
PSNF	0.73 (0.20)	0.18 (0.17)	0.82 (0.12)	0.49 (0.24)	0.18 (0.51)	0.42 (0.22)
NSFF	0.82 (0.17)	0.10 (0.15)	0.89 (0.07)	0.64 (0.19)	0.23 (0.52)	0.40 (0.23)
NSHF	0.79 (0.18)	0.21 (0.23)	0.83 (0.11)	0.51 (0.24)	0.03 (0.56)	0.48 (0.24)
NSNF	0.78 (0.19)	0.16 (0.15)	0.85 (0.10)	0.55 (0.22)	0.12 (0.50)	0.45 (0.21)

*GS, grisly scene; HS, pleasant scene; NS, neutral scene; FF, fearful face; HF, happy face; NF, neutral face.*

A similar ANOVA showed no effect of emotional face on $B_{D}^{{}^{\prime \prime }}$, [*F* (2, 78) = 2.09, *p* = 0.14, η*_p_*^2^ = 0.051] or *Br* [*F* (2, 78) = 1.32, *p* = 0.27, η*_p_*^2^ = 0.033], and no interaction between scene and emotional face for both variables [*F* (4, 156) = 0.68, *p* = 0.61, η*_p_*^2^ = 0.017; and *F* (4, 156) = 0.83, *p* = 0.51, η*_p_*^2^ = 0.021, respectively]. Simple effects analysis showed no effect of scene on $B_{D}^{{}^{\prime \prime }}$ [*F* (2, 78) = 0.71, *p* = 0.47, η*_p_*^2^ = 0.018] or on *Br* [*F* (2, 78) = 0.68, *p* = 0.49, η*_p_*^2^ = 0.017].

#### Gaze Tracking Data

**FIGURE 3:**
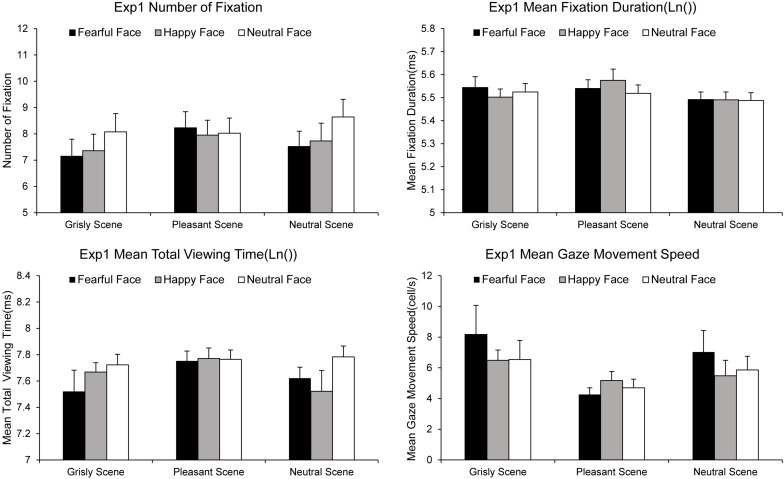
Gaze movements results for experiment 1. Error bars represent standard errors of the mean (see Supplementary Material for datasets).

The NF, MFD, MTVT, and SPEED values computed for each experimental condition are summarized in Figure 3. A 3 × 3 ANOVA [(scene: pleasant vs. grisly vs. neutral) × (emotional face: happy vs. fearful vs. neutral)] showed a significant main effect of emotional face on NF [*F* (2, 78) = 3.49, *p* = 0.035, η*_p_*^2^ = 0.08] and MTVT [*F* (2, 78) = 2.96, *p* = 0.058, η*_p_*^2^ = 0.07], suggesting that the number of fixations and participants’ total viewing times were greater for neutral faces than for fearful and happy faces in both scenes. An ANOVA of the MFD revealed a main effect of scene [*F* (2, 78) = 3.97, *p* = 0.023, η*_p_*^2^ = 0.09], indicating that the MFD was shorter for the pleasant scene than for the neutral scene [difference_neutral – pleasant_ = −0.055, *p* = 0.002, SE = 0.016, 95% CI = (−0.088, −0.022)]. An ANOVA of the SPEED revealed a marginally significant main effect of emotional face [*F* (2, 78) = 4.23, *p* = 0.023, η*_p_*^2^ = 0.10], suggesting that the SPEED was lower for happy faces than for fearful and neutral faces.

**FIGURE 4:**
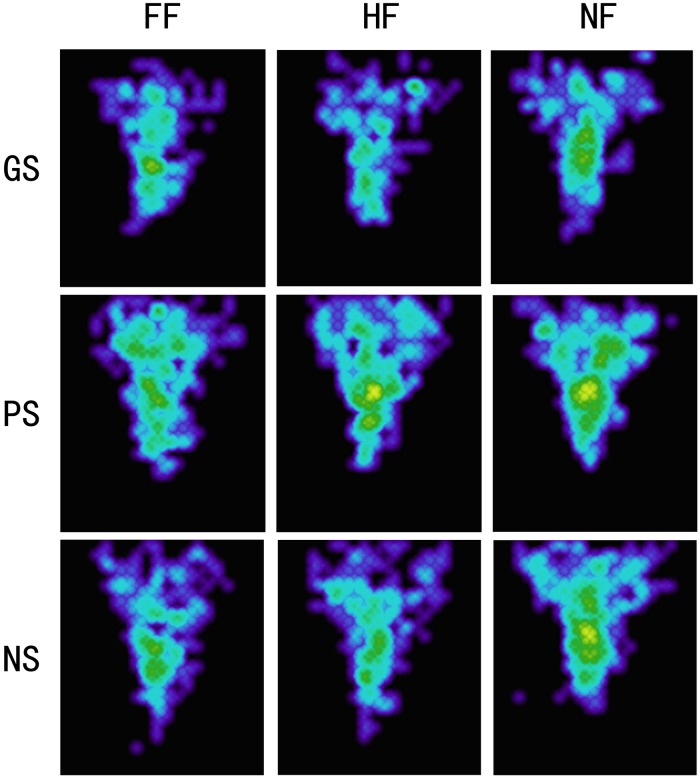
Heat maps for experiment 1. GS, grisly scene; HS, pleasant scene; NS, neutral scene; FF, fearful face; HF, happy face; NF, neutral face.

Attention patterns indicated by heat maps of the fixation areas for each experimental condition are presented in Figure 4. The fixation areas under four experimental conditions were computed for each participant. A 3 × 3 ANOVA [(scene: pleasant vs. grisly vs. neutral) × (emotional face: happy vs. fearful vs. neutral)] showed a main effect of scene [*F* (2, 78) = 4.49, *p* = 0.021, η*_p_*^2^ = 0.10] and a main effect of emotional face [*F* (2, 78) = 4.86, *p* = 0.022, η*_p_*^2^ = 0.11]. A pairwise comparison analysis indicated that fixation areas were smaller for the grisly scene than for the pleasant and neutral scenes [difference_pleasant – grisly_ = 0.81, *p* = 0.021, SE = 0.34, 95% CI = (0.13, 1.49); difference_neutral – grisly_ = 0.46, *p* = 0.022, SE = 0.20, 95% CI = (0.07, 0.86)] and also revealed a relatively narrower and more concentrated attentional pattern for fearful and happy faces than for neutral faces, as indicated by the fixation areas [difference_neutral – fearful_ = 0.39, *p* = 0.018, SE = 0.16, 95% CI = (0.07, 0.71); difference_neutral – happy_ = 0.35, *p* = 0.035, SE = 0.16, 95% CI = (0.03, 0.67)]. Compared to fearful face, happy face led to a relatively broad fixation area, but the difference was insignificant [difference_happy – fearful_ = 0.04, *p* = 0.703, SE = 0.10, 95% CI = (−0.17, 0.25)].

### Discussion

The findings of experiment 1 were similar to those of pilot experiments. Naturally induced moods failed to cause mood-congruent effects but did lead to a mood effect independent of a strong emotional face effect. The results of the experiment confirm the hypothesis that mood-congruent effects are difficult to observe with natural moods, especially when emotional pictures are concerned. In other words, emotional scenes might exert an impact on the learning of emotional stimuli but do not necessary interact with the emotional content of these stimuli.

Gaze tracking recordings in the experiment showed consistently that happy faces caused more fixations yet with a shorter fixation duration and that the grisly scene led to faster gaze movements compared to the pleasant scene. Together with memory tests, gaze tracking recordings in the experiment provide direct evidence for the emotional face effect and scene effect at the level of affect-driven attention. Although gaze tracking failed to predict recognition memory tests, it validated the observation of mood effects in cognitive processing.

Although a mood effect was observed in experiment 1, it was hard to rule out the possibility that the unmatched arousal level of the induced moods played a role. In addition, a strong emotional face effect might attenuate the potential mood effect. To obtain a well-controlled mood induction, we conducted experiment 2, in which pleasant and grisly scenes were carefully matched on both valence and arousal levels. In addition, emotional faces were replaced with neutral faces to observe a pure mood effect.

## Experiment 2

In this experiment, a pleasant scene and a grisly scene were used, and the classic recognition memory task was performed with only neutral faces to explore further the possible mood effects on neutral face recognition memory.

### Methods

#### Participants and Apparatus

A sample of 102 participants recruited from courses in experimental psychology took part in experiment 2 in return for course credits (13 male, 89 female; mean age, 19.85 ± 1.00 years; age range, 18–27 years). The experiment was performed using a more portable apparatus, which consisted of the following: (1) a head-mounted virtual reality helmet (HTC VIVE, HTC Corporation, with a 110° horizontal field of view; resolution, 1,080 × 1,200 pixels per eye; refresh rate, 90 Hz; integrated microphone, and controller specs); (2) an infrared tracking subsystem (3.5 × 3.5 m tracking area; refresh rate, 100 Hz); and (3) an HP workstation (CPU E5-2667 3.20 GHz, RAM 56.0 GB, GPU NVIDIA Quadro K6000). The virtual reality environment (VRE) was presented on the head-mounted helmet, which was tracked by the infrared tracking subsystem.

#### Materials

##### VREs

Three VREs were designed: neutral, pleasant, and grisly. The neutral VRE was an empty scene with beige walls intended to buffer moods before exposure to a positive mood (pleasant scene) or negative mood (grisly scene).

For the pleasant VRE, a garden was created in the hollow square learning area with red brick perimeter walls and a blue skybox over the area. The garden had a rich variety of beautiful flowers, while timber palisades were arranged along a grass garden path in the hollow square corridor. There was a beautiful big tree at each corner of the corridor, and several stones were placed under the trees, as shown in Figure 1. Rose petals drifted down from the sky as the song “Spring Sonata” (allegro) played.

For the grisly VRE, the red brick walls were replaced with drab gray walls. The four trees were made invisible with spider webs emanating from their locations and the walls. Withered grass, tombstones, black iron railings, and skulls were arranged along the path in place of flowers, timber palisades, and stones. Several owls stood on the walls, iron railings and tombstones, and some gray-colored rats with one red eye moved about on the floor. Beethoven’s Symphony No. 3 (a funeral march) played, and hell money (the money used by ghosts in the hell in Chinese folktales) fell from the sky instead of rose petals. Notably, most high-fidelity models were revised to be low-fidelity models to reduce arousal. In addition, low-fidelity models were suitable for a mobile workstation computer.

##### Face image

All 96 faces used as stimuli were photos of neutral faces selected from a database created by our laboratory. In contrast to experiment 1, all neutral faces were of the same size (300 × 350 pixels) and used color. The valence and arousal assessments are shown in Table 3.

**TABLE 3:** Assessments of faces and scene in experiment 2 (SD) and emotional face recognition results for experiment 2 (SD).

Parameter	Face				Scene			
		
	Female		Male		Grisly		Pleasant	
Valence	5.54 (1.06)		4.84 (1.01)		3.70 (1.35)		4.93 (1.23)	
Arousal	4.17 (0.84)		3.94 (0.62)		4.22 (1.35)		4.24 (1.44)	

**Condition**	**Raw measure**		**Discrimination**			**Bias**		
			
	**Hit rate**	**FA rate**	***A*′**	***Pr***		$B_{D}^{{}^{\prime \prime }}$		***Br***

GSNF	0.65 (0.18)	0.12 (0.10)	0.84 (0.09)	0.51 (0.21)		0.50 (0.37)		0.29 (0.17)
PSNF	0.67 (0.18)	0.11 (0.10)	0.85 (0.09)	0.53 (0.20)		0.52 (0.36)		0.27 (0.17)

*Faces were assessed on a 9-point Likert scales pre-experiment; scene was evaluated on 7-point Likert scales during experiment. GSNF, grisly scene and neutral face; PSNF, pleasant scene and neutral face (see Supplementary Material for datasets).*

#### Procedure and Design

##### Procedure

The whole experiment consisted of a questionnaire phase, a learning phase, and a testing phase. In the questionnaire phase, participants were asked to answer two questions by telephone to rule out serious anxiety problems or rodent phobia. When they came to the laboratory, they were asked to complete an SDS questionnaire to rule out depression. Mood was assessed during the learning and testing phases. All participants were told that they could ask for a pause and withdraw from the experiment if they felt uncomfortable.

In the learning phase, participants were instructed to practice walking along the hollow square corridor to experience a scene. They were then asked to walk up to the face they saw in the corridor. They were encouraged to pause in front of the face for a while to study it. They pressed the left bumper button of a wireless joystick to let the face disappear when they felt that they had memorized each face. To ensure sufficient learning time, there was a 500-ms minimal accumulated target-face fixation time before the left bumper button became functional. After learning a full set of eight faces, the participants would have completed a full walk around the corridor. A testing room door then appeared at the end of the corridor, and the participants were instructed to step into the room for the testing phase.

After entering the testing room, participants were instructed to press the left bumper button to close the testing room door and end the music. Immediately thereafter, two 7-point scales appeared on the testing room wall one at a time. Participants reported the valence and arousal level they had just felt in the corridor by selecting a number button on the wall. A random number then appeared, and participants were asked to subtract 7 from it, report the result loudly, and then continue to subtract 7 from the result just reported. The loop calculation lasted for 30 s. A face recognition test followed the calculation, in which the participants were instructed to recognize the face appearing on the wall. They initiated the test by pressing the left bumper button, after which the test began with a 500-ms fixation cross. Subsequently, a face was presented on the wall for a maximum of 1,000 ms. It disappeared when the participant pressed the “X” or “B” button to identify a learned or new face, respectively. All 16 faces (8 learned and 8 new) were randomly displayed, and the responses were recorded. The procedure was identical for all blocks.

##### Design

The experiment was based on a one-factor within subject design (scene: grisly vs. pleasant). The scene order (pleasant then grisly or vice versa) was pseudo-random across subjects. Each scene was presented in two consecutive blocks in which 12 target faces were prearranged in the hollow square corridor. At the end of each block, there was a face recognition test.

### Results and Discussion

The mood assessment results for the pleasant and grisly scenes are shown in Table 3. Paired samples tests showed that valence was rated lower for the grisly scene than for the pleasant scene [*t* (101) = −8.75, *p* < 0.001, *d* = 0.87] and that arousal was balanced for the two scenes [*t* (101) = −0.145, *p* = 0.885, *d* = 0.01]. These results showed that each VRE scene was able to induce its intended mood.

The results for discriminability are shown in Table 3. A one-factor repeated ANOVA showed no difference in hit rate [*F* (1, 101) = 0.62, *p* = 0.43, η*_p_*^2^ = 0.006] or FA [*F* (1, 101) = 2.51, *p* = 0.12, η*_p_*^2^ = 0.02]. Similar ANOVA on discriminability also showed no difference in *A*′ [*F* (1, 101) = 1.98, *p* = 0.16, η*_p_*^2^ = 0.02]; in *Pr* [*F* (1, 101) = 2.26, *p* = 0.14, η*_p_*^2^ = 0.02]; in$B_{D}^{{}^{\prime \prime }}$, [*F* (1, 101) = 0.33, *p* = 0.57, η*_p_*^2^ = 0.003]; or in *Br* [*F* (1, 101) = 0.54, *p* = 0.46, η*_p_*^2^ = 0.005].

Mood induction via VREs was confirmed again. The results of experiment 2 revealed the absence of a mood effect when the valence and arousal levels were matched between the pleasant and grisly scenes.

## General Discussion

The aim of the present study was to examine whether mood-congruent effect could be observed with natural moods, how real-time VR-based natural moods interact with emotional face recognition, and how the valence and arousal levels of VR-based natural moods can be controlled for mood-related research. Experiment 1 failed to demonstrate a mood-congruent effect in a classic face recognition task, although immediate mood assessments made by participants confirmed that the natural mood induction via VREs had been successful. These results are in line with a previous study of natural moods (Hasher et al., 1985). Instead of interaction, experiment 1 demonstrated a mood effect independent of a strong emotional face effect. Gaze tracking results provide further evidence for the emotional face and mood effects. More importantly, the manipulations of valence and arousal levels of the induced moods in the present study revealed that valence and arousal levels both played a role in the mood effect. Experiment 2 further revealed that mood effect diminished when valence and arousal levels were both comparable across grisly and pleasant scenes. The findings of the current study suggest that controlling valence and arousal levels is a necessary challenge in future mood effect research (Parrott and Sabini, 1990; Mayer et al., 1995; Forgas et al., 2009; Farach et al., 2014).

With the manipulations of both valence and arousal levels, the present study showed that neither valence nor arousal could account for all of the demonstrated mood effects by themselves. Both valence- and arousal-based interpretations should be taken into consideration since the findings on mood effects are susceptible to manipulations of mood induction. In the present study, experiment 1 demonstrated a mood effect by improving the valence of the pleasant scene used in pilot experiments. Similarly, the mood effect disappeared in experiment 2 when arousal levels were balanced between happy and fearful moods. The SPEED of gaze movement provide further evidence for such a valence and arousal effect in that mood effect was manifestly observed in experiment 1. These results are in line with the findings that saccadic velocity reflects the level of arousal and activation (Galley, 1989; App and Debus, 1998; Di Stasi et al., 2010; Di Stasi et al., 2013). Additionally, the Yerkes–Dodson law, as revealed in the interaction between arousal level and cognitive performance, and the finding of a positive-negative asymmetry in valence (Westermann et al., 1996; Kensinger, 2007; Becker et al., 2010; Alves et al., 2015), have both predicted that mood effects are essentially related to both valence and arousal levels of the induced moods. No correlations were found in the current study between arousal level and face discriminability individually, but the control of valence and arousal did demonstrate a mood effect across the two experiments.

Although the present study failed to detect a mood-congruent effect with natural moods, it introduced a new method that allows more manipulation in natural mood induction. In future research, a well-controlled mood induction combined with direct observation of emotional attention would be practicable and valuable for many of the important problems in this field. In VR-based mood induction, valence and arousal can be manipulated easily by adding or removing fantastic, fearful, or other eliciting stimuli. Several studies have shown that a mood induced in a VRE is comparable to a real natural mood for its typical features of immersion, imagination, and interaction (Felnhofer et al., 2015; Cao et al., 2017). Notably, ways to control valence and arousal levels usually overlap but are essentially different. For example, we used low-fidelity models in experiment 2 to reduce the arousal level of the grisly scene. The evidence from our pilot experiments suggests that valence control can be reached by adding or removing eliciting objects, whereas arousal control is mainly dependent on high-quality simulation. Therefore, an obvious limitation arises from the relatively low quality of the helmet display. With technological developments, VR-based mood induction could become an excellent method of induction.

In the current study, a robust negativity bias in emotional face recognition was found to accompany a mood repair effect (Sanchez et al., 2014) in gaze tracking. Fearful faces were better discriminated than happy faces under all experimental conditions, and FA rates were lower for fearful faces than for happy faces, which is in line with previous studies (Storbeck and Clore, 2005; Kensinger, 2007). Like the attentional bias toward threat-related stimuli, evolutionary and biological bases could be used to explain such a negativity effect (Juth et al., 2005). Surprisingly, our NF gaze tracking results showed a tendency for participants to have more fixations on happy faces and spend longer viewing them compared to fearful faces, although the fearful faces were better encoded. These results are consistent with a protective bias (Biss et al., 2010; Newman and Sears, 2015) and support the notion of mood repair (Sanchez et al., 2014). Heat maps of fixation data have also shown a tendency for participants to broaden their scope of fixation on happy faces compared to fearful faces (Wadlinger and Isaacowitz, 2006). Studies examining threatening face recognition have suggested that a self-protective motivation may enhance encoding efficiency for threatening faces (Becker et al., 2010; Van Bockstaele et al., 2014). In the present study, fearful faces appeared to trigger such a self-protection mode, which not only enhanced fearful face encoding but also biased attention toward happy faces.

The current study provides novel and interesting data with regard to mood congruity research, but several limitations must be noted. First, although the within-subject design helped to control for individual differences, it might have attenuated a mood effect due to interference between positive and negative mood inductions. Second, there is a possibility that real-time mood induction might differ from pre-experimental mood induction, an issue that could be explored further in future study. Finally, there is a possibility that mood-congruent effects might be found when arousal levels are perfectly balanced in typical pleasant and grisly scenes. Such a valence and arousal match would be difficult to achieve yet worth the attempt in future research.

To conclude, the findings of the present study can be summarized as: (a) mood-congruent recall (or mood-incongruent recall could not be observed with natural moods), (b) valence and arousal levels of the induced moods both contributed to the mood effect, and (c) VR-based eye-tracking recording is a promising method for future mood and emotion research.

## Data Availability Statement

All datasets generated for this study are included in the article/Supplementary Material.

## Ethics Statement

The studies involving human participants were reviewed and approved by the Institutional Review Board of Capital Normal University. The patients/participants provided their written informed consent to participate in this study.

## Author Contributions

LZ, YW, HK, and JD conceived and designed the experiments. LZ developed the VREs, wrote all the program, and performed the experiments. LZ and YW analyzed the data and wrote the manuscript.

## Conflict of Interest

The authors declare that the research was conducted in the absence of any commercial or financial relationships that could be construed as a potential conflict of interest.
